# Wds-Mediated H3K4me3 Modification Regulates Lipid Synthesis and Transport in *Drosophila*

**DOI:** 10.3390/ijms24076125

**Published:** 2023-03-24

**Authors:** Tujing Zhao, Min Wang, Zheng Li, Hao Li, Dongqin Yuan, Xing Zhang, Mengge Guo, Wenliang Qian, Daojun Cheng

**Affiliations:** 1 State Key Laboratory of Silkworm Genome Biology, Biological Science Research Center, Southwest University, Chongqing 400715, China; 2 Chongqing Key Laboratory of Sericultural Science, Southwest University, Chongqing 400715, China

**Keywords:** H3K4me3, Wds, Hsf, lipid synthesis, lipid transport, fat body, intestine

## Abstract

Lipid homeostasis is essential for insect growth and development. The complex of proteins associated with Set 1 (COMPASS)-catalyzed Histone 3 lysine 4 trimethylation (H3K4me3) epigenetically activates gene transcription and is involved in various biological processes, but the role and molecular mechanism of H3K4me3 modification in lipid homeostasis remains largely unknown. In the present study, we showed in *Drosophila* that fat body-specific knockdown of *will die slowly* (*Wds*) as one of the COMPASS complex components caused a decrease in lipid droplet (LD) size and triglyceride (TG) levels. Mechanistically, Wds-mediated H3K4me3 modification in the fat body targeted several lipogenic genes involved in lipid synthesis and the *Lpp* gene associated with lipid transport to promote their expressions; the transcription factor heat shock factor (Hsf) could interact with Wds to modulate H3K4me3 modification within the promoters of these targets; and fat body-specific knockdown of *Hsf* phenocopied the effects of *Wds* knockdown on lipid homeostasis in the fat body. Moreover, fat body-specific knockdown of *Wds* or *Hsf* reduced high-fat diet (HFD)-induced oversized LDs and high TG levels. Altogether, our study reveals that Wds-mediated H3K4me3 modification is required for lipid homeostasis during *Drosophila* development and provides novel insights into the epigenetic regulation of insect lipid metabolism.

## 1. Introduction

Lipids are important cellular components that play key roles in insect energy homeostasis [1,2,3]. During the feeding stage in insects, large amounts of lipids that are stored in the fat body, a tissue that is equivalent to vertebrate adipose tissue and liver, controls energy storage and mobilization to supply the energy for growth, development, and other biological processes [4,5,6,7,8,9].

In insect fat body, lipids are generally derived from de novo lipogenesis or dietary fatty acids from the intestine and stored as neutral lipids, namely triglycerides (TG). For de novo lipogenesis (de novo fatty acid synthesis), acetyl-CoA from glucose catabolism is first catalyzed into long-chain fatty acids via two carboxylation steps mediated by acetyl-CoA carboxylase [10] and fatty acid synthase (FASN1) [1,11]. Then, these newly de novo synthesized fatty acids are activated to FA-CoA by Acyl-CoA synthetase long-chain (Acsl) and next esterified to form the TG by consecutive esterification reactions catalyzed by several lipogenic enzymes, including phosphatidylate phosphatase (Lipin) and diacylglycerol O-acyltransferase (DGAT) encoded by the midway (Mdy) gene [12,13]. Previous reports have shown that the loss of lipogenic genes results in lipid synthesis defects, developmental disorders, and lower survival rates [7,14,15,16]. Except for de novo synthesis, the transport of dietary lipids is another important process for TG formation in the fat body. After being digested by lipase in the intestine, dietary lipids are absorbed by enterocytes and packaged into lipoprotein particles by lipoprotein Lipophorin (Lpp) [17,18]. Lpp is produced in the fat body and functions as the major hemolymph lipid carrier to transport lipids into the fat body for synthesizing TG [18]. All synthesized TG is ultimately stored in lipid droplets (LD), lipid centers and energy homeostasis organelles consisting of a central hydrophobic core of most TG and sterol esters [1,2,19]. Moreover, a number of transcription factors have been identified to be involved in the regulation of lipid homeostasis, such as sterol regulatory element-binding protein (SREBP), Forkhead box O (FOXO), TATA-box binding protein (TBP), and some nuclear receptors [20,21,22,23,24,25]. However, the mechanism underlying transcriptional regulation of insect lipid homeostasis remain poorly understood.

Histone 3 lysine 4 trimethylation (H3K4me3) is an epigenetic modification that is catalyzed by the complex of proteins associated with Set 1 (COMPASS) and has been characterized as an active mark that is closely associated with facilitating gene transcription to regulate various biological processes [26,27]. Several studies have previously indicated that H3K4me3 modification is epigenetically associated with lipid metabolism. For example, H3K4me3 in human adipose tissue shows a correlation with the expressions of genes involved in lipid metabolism and inflammation [28]. Histone H3K4 methyltransferase MLL3 modulates the adipogenesis of white fat in mice [29,30,31,32]. Recent work in *Caenorhabditis elegans* revealed that high H3K4me3 levels cause a transgenerational epigenetic inheritance of obesogenic effects by upregulating SREBP transcription [33]. Collectively, the molecular mechanism underlying H3K4me3 regulation of lipid metabolism remains to be deeply deciphered.

In this study, we uncovered in *Drosophila* that H3K4me3 modification is transcriptionally involved in regulating TG synthesis in the fat body and lipid transport from the intestine to the hemolymph. We showed that fat body-specific knockdown of *will die slowly* (*Wds*), a gene encoding Wds protein as a core subunit of the COMPASS complex, resulted in a reduction in LD size and TG synthesis in the fat body as well as in lipid transport from the intestine. Mechanistically, a conjoint analysis of RNA-sequencing (RNA-seq) and chromatin immunoprecipitation sequencing (ChIP-seq) data identified that *Wds* knockdown in the fat body downregulated the expressions of genes involved in lipogenesis and lipid transport by blocking H3K4me3 deposition within their promoters. Wds physically interacted with the transcription factor heat shock factor (Hsf) that modulates the transcriptions of genes associated with lipogenesis and lipid transport. High-fat diet (HFD) feeding promotes the transcription of *Wds* and *Hsf* and increases global H3K4me3 levels in the fat body. Together, our data revealed that Wds-mediated H3K4me3 modification epigenetically regulates lipid homeostasis in response to HFD condition in *Drosophila*.

## 2. Results

### 2.1. Disrupting Wds-Mezdiated H3K4me3 Modification in the Drosophila Fat Body Reduced Lipid Content

**Figure 1:**
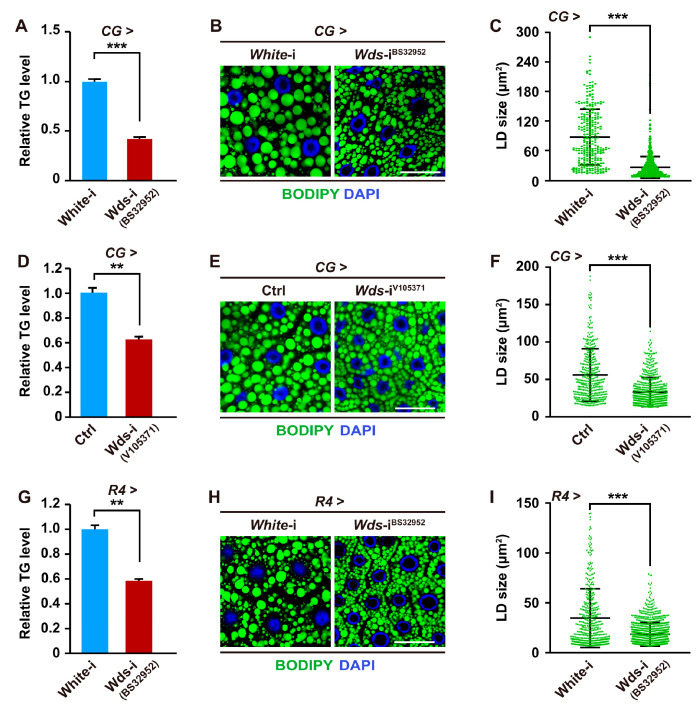
*Wds* knockdown in the *Drosophila* fat body impaired lipid content. (**A**–**C**) TRiP RNAi line (BS32952)-mediated *Wds* knockdown of *Drosophila* larvae using CG-Gal4 decreased the TG levels (n = 3, 10 larvae per group) of third instar larvae (**A**). This genetic manipulation for *Wds* was used hereafter unless otherwise indicated. Fat body-specific *Wds* knockdown decreased the LD size in the fat body of third instar larvae (**B**,**C**). Quantification of LDs size (**C**). Each point represents a single LD. BODIPY, green; DAPI, blue. Scale bar, 50 μm. (**D**–**F**) VDRC RNAi line (V105371)-mediated *Wds* knockdown in the fat body using CG-Gal4 decreased TG levels (n = 3, 10 larvae per group) of third instar larvae (**D**) and reduced LD size (**E**,**F**) in the fat body of *Drosophila* third instar larvae. V60100 line was used as control. (**G**–**I**) TRiP RNAi line (BS32952)-mediated *Wds* knockdown in the fat body using R4-Gal4 decreased TG levels (n = 3, 10 larvae per group) of third instar larvae (**G**) of *Drosophila* larvae and reduced LD size (**H**,**I**) in the fat body. Each point represents a single LD (**H**). BODIPY, green; DAPI, blue. Scale bar, 50 μm. Data are presented as the mean ± SE (error bars). For the significance: ** *p* < 0.01 and *** *p* < 0.001 versus the control.

To test whether H3K4me3 modification is involved in insect lipid metabolism, we conducted fat body-specific RNAi-mediated knockdown of the *Drosophila Wds* gene, which encodes a COMPASS component that functions as an effector protein to link the complex to the H3 tail and enhances methyltransferase activity [34]. We first performed *Wds* knockdown in the fat body of *Drosophila* larvae using the fat body-specific CG-Gal4 driver. The results showed that fat body-specific knockdown of the *Wds* gene, using a TRiP RNAi line (BS32952; this line and CG-Gal4 were used for genetic manipulation of *Wds* hereafter unless otherwise indicated) and a VDRC RNAi line (V105371) that target distinct sequences, led to a dramatical reduction in larvae TG levels and LD size in the larval fat body (Figure 1A–F and Figure S1A,B). As expected, the global H3K4me3 levels in the fat body also were decreased in *Wds* knockdown larvae (Figure S2A,B). In addition, the knockdown of *Wds* by using R4-Gal4, another fat body-specific driver, also caused an abnormality in TG levels and LD size (Figure 1G–I), phenocopying the effects of CG-Gal4-driven *Wds* knockdown. Collectively, our data demonstrate that Wds-mediated H3K4me3 modification in the *Drosophila* fat body is positively involved in lipid homeostasis.

### 2.2. Fat Body-Specific Wds Knockdown Impaired the Transcription of Lipogenic Genes by Reducing H3K4me3 Deposition within Their Promoters

**Figure 2:**
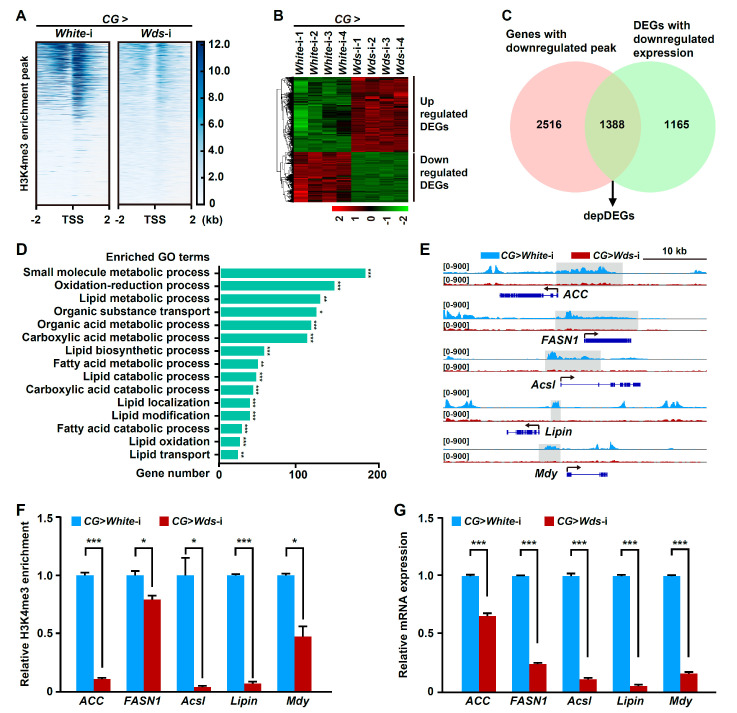
Fat body-specific *Wds* knockdown reduced H3K4me3 deposition within the promoters of lipogenic genes and impaired their transcriptions. (**A**) ChIP-seq identified the change of H3K4me3 enrichment around genome-wide transcription start sites (TSSs) in the fat body of *Drosophila* third instar larvae following fat body-specific *Wds* knockdown. The heatmap of H3K4me3 ChIP peaks around the TSSs was constructed. (**B**) Hierarchical clustering of all differentially expressed genes (DEGs) induced by *Wds* knockdown in the fat body. (**C**) RNA-seq-based identification of differentially expressed genes with downregulated mRNA expression and downregulated H3K4me3 ChIP peaks (depDEGs) after fat body-specific *Wds* knockdown. (**D**) GO enrichment of depDEGs after fat body-specific *Wds* knockdown. (**E**) Fat body-specific *Wds* knockdown-caused changes in H3K4me3 ChIP peaks within the promoters of several lipogenic genes from the depDEGs, including *ACC*, *FASN1*, *Acsl*, *Lipin*, and *Mdy*. The representative peaks were highlighted in grey. Arrows indicate the direction of gene transcription. (**F**) ChIP-qPCR confirmation of fat body-specific *Wds* knockdown-caused decrease in H3K4me3 enrichment at lipogenic genes in the fat body of *Drosophila* third instar larvae. (**G**) RT-qPCR confirmation of fat body-specific *Wds* knockdown-caused downregulation in mRNA expression of lipogenic genes in the fat body (n = 3, 10 larvae per group). Data are presented as the mean ± SE (error bars). For the significance: * *p* < 0.05, ** *p* < 0.01, and *** *p* < 0.001 versus the control.

H3K4me3 modification is an active epigenetic marker. To understand the regulatory mechanism underlying H3K4me3 regulation of lipid metabolism in the *Drosophila* fat body, we sought to identify direct targets of H3K4me3 modification by performing chromatin immunoprecipitation sequencing (ChIP-seq) using anti-H3K4me3 antibodies and RNA sequencing (RNA-seq) in the fat body of *Drosophila* larvae with *Wds* knockdown and the control. First, ChIP-seq data showed that more than 60% of ChIP peaks were located within the promoter regions (<= 2000 bp upstream of the transcription start sites, TSS) (Figure S3A,B), and H3K4me3 signals near TSS were significantly decreased following fat body-specific *Wds* knockdown (Figure 2A and Figure S3C). Further comparative analysis identified that among 7806 differential H3K4me3 ChIP peaks, 4149 peaks that were located within the promoter regions were downregulated in the fat body with *Wds* knockdown compared to the control and were associated with 3904 genes (Tables S1–S3). Further RNA-seq analysis identified 5704 differentially expressed genes (DEGs) in the fat body between *Drosophila* larvae with fat body-specific *Wds* knockdown and the control, 2553 of which were downregulated following *Wds* knockdown (Figure 2B, Figure S3D, and Table S4). Importantly, a joint comparative analysis identified that 1388 downregulated DEGs (referred to as depDEGs) were included in the collection of genes with downregulated H3K4me3 ChIP peaks in their promoters (Figure 2C and Table S5), and these genes were enriched in the lipid biosynthetic and fatty acid metabolic processes by Gene Ontology (GO) annotation (Figure 2D). Encyclopedia of Genes and Genomes (KEGG) pathway enrichment analysis showed that depDEGs also were involved in glycolysis, glycerolipid metabolism, and fatty acid biosynthesis (Figure S3E). 

Notably, we found that several lipogenic genes, including *ACC*, *FASN1*, *Acsl*, *Lipin*, and *Mdy*, were included in this depDEGs collection and had H3K4me3 ChIP peaks within their promoters (Figure 2E). Further ChIP-PCR assays and ChIP-qPCR confirmed H3K4me3 deposition within the promoters of these lipogenic genes, and this deposition could be downregulated by fat body-specific *Wds* knockdown (Figure 2F and Figure S4A–E). Consistently, RNA-seq analysis and following RT-qPCR assay showed that the expressions of these lipogenic genes were decreased in the fat body following fat body-specific *Wds* knockdown (Figure 2G and Figure S3F). Taken together, these results indicate that Wds-mediate H3K4me3 positively modulates fatty acid synthesis and TG formation by targeting the lipogenic genes to promote the expression. 

### 2.3. Wds-Mediated H3K4me3 Modification Regulates Lipid Transport from Intestine by Modulating the Expression of the Lpp Gene in the Fat Body

**Figure 3:**
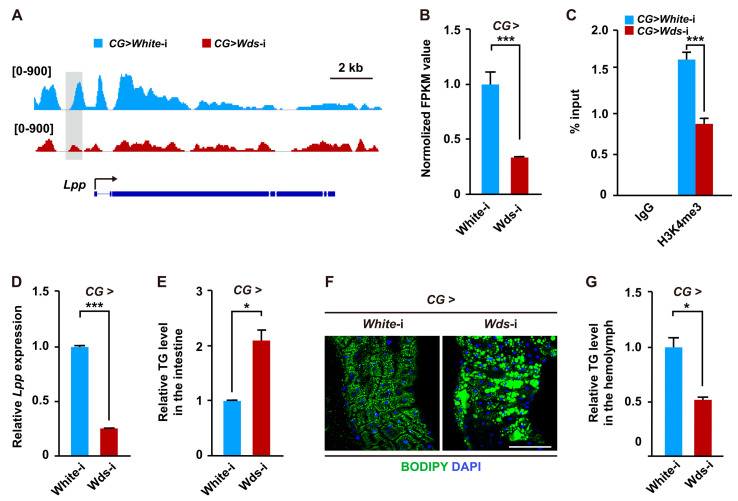
Fat body-specific knockdown of *Wds* downregulated *Lpp* transcription and impaired lipid transport. (**A**) Fat body-specific *Wds* knockdown-caused changes in H3K4me3 ChIP peaks within the promoter of the *Lpp* gene that encodes a lipoprotein mediating lipid transport from the intestine and was also included in the depDEGs. The representative peak was highlighted in grey. The arrow indicated transcription direction. (**B**) RNA-seq data of *Wds* knockdown-induced decrease in *Lpp* mRNA expression in the larval fat body. FPKM, fragments per kilo base of transcript per million mapped fragments. (**C**) ChIP-qPCR confirmation of fat body-specific *Wds* knockdown-caused decrease in H3K4me3 enrichment within the *Lpp* gene in the fat body of *Drosophila* third instar larvae. (**D**) *Wds* knockdown in the fat body downregulated *Lpp* transcription in the fat body (n = 3, 10 larvae per group). (**E**,**F**) Fat body-specific *Wds* knockdown caused elevated TG levels (n = 3, 20 larvae per group) (**E**) and lipid accumulation (**F**) in the third instar larvae intestine but decreased TG levels (n = 3, 60 larvae per group) in the hemolymph (**G**). BODIPY, green; DAPI, blue. Scale bar, 100 μm. Data are presented as the mean ± SE (error bars). For the significance: * *p* < 0.05 and *** *p* < 0.001 versus the control.

We also noted that several genes involved in lipid transport were downregulated in the fat body of *Drosophila* larvae with fat body-specific *Wds* knockdown (Figure 2C,D and Table S5), suggesting that Wds-mediated H3K4me3 modification may regulate lipid transport. We therefore focused on the *Lpp* gene, because it encodes a main apolipoprotein of the ApoB family and is generally secreted from the fat body and then recruited to the intestine to transport dietary lipids [18]. Our data showed that compared to the control, H3K4me3 enrichment within the promoter of the *Lpp* gene was dramatically impaired following fat body-specific *Wds* knockdown (Figure 3A). ChIP-PCR assays in the fat body of *Drosophila* larvae further respectively confirmed that the region covering the H3K4me3 ChIP peak within the *Lpp* promoter could be immunoprecipitated by an anti-H3K4me3 antibody (Figure S4F). RNA-seq analysis showed that *Lpp* mRNA expression was also downregulated in *Wds* knockdown *Drosophila* larvae (Figure 3B). Moreover, ChIP-qPCR assay and RT-qPCR examination respectively confirmed that fat body-specific *Wds* knockdown dramatically impaired the H3K4me3 enrichment within the promoters of *Lpp* genes and diminished its transcription in the fat body (Figure 3C,D). These results demonstrate that Wds-mediated H3K4me3 modification targets the *Lpp* gene to promote its transcription.

Furthermore, we investigated the effect of fat body-specific *Wds* knockdown on lipid transport from the intestine in *Drosophila*. The results showed that *Wds* knockdown in the fat body significantly increased TG levels in the intestine (Figure 3E). In addition, we also observed an obvious lipid accumulation in the intestine following fat body-specific *Wds* knockdown compared to the control (Figure 3F), but the TG levels in the hemolymph were decreased (Figure 3G). Collectively, our results indicate that Wds-mediated H3K4me3 modification in the fat body positively regulates *Lpp* expression to control lipid transport from the intestine.

### 2.4. Transcription Factor Hsf Promotes Lipid Synthesis and Transport by Interacting with Wds to Modulate H3K4me3 Modification 

**Figure 4:**
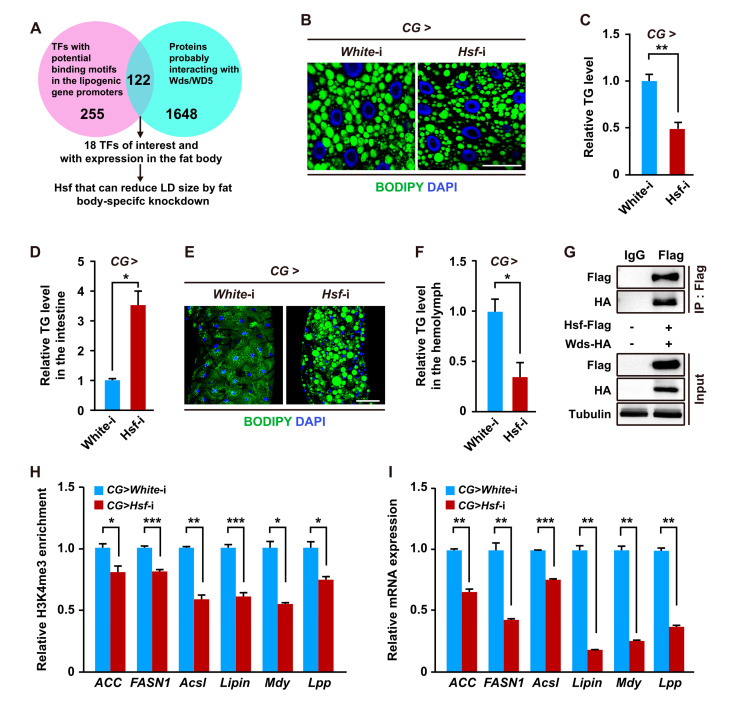
Hsf promoted lipid synthesis and transport by interacting with Wds to modulate H3K4me3 modification. (**A**) Schematic diagram for multiple approaches-based identification of the transcription factor Hsf that might interact with Wds, had potential binding motifs within the promoters of lipid homeostasis-related genes, and was involved in *Drosophila* lipid synthesis. (**B**,**C**) TRiP RNAi line (THU2458)-mediated *Hsf* knockdown in the *Drosophila* fat body reduced LD size (**B**) and decreased the TG levels (n = 3, 10 larvae per group) (**C**) of *Drosophila* third instar larvae. BODIPY, green; DAPI, blue. Scale bar, 50 μm. (**D**–**F**) Fat body-specific *Hsf* knockdown caused elevated TG levels (n = 3, 20 larvae per group) (**D**) and lipid accumulation (**E**) in the intestine but decreased TG levels (n = 3, 60 larvae per group) in the hemolymph (**F**). BODIPY, green; DAPI, blue. Scale bar, 100 μm (**E**). (**G**) Co-IP confirmation of the interaction between Flag-tagged Hsf and HA-tagged Wds that were transiently expressed in *Drosophila* S2 cells. The antibodies against the HA tag or Flag tag were used. Tubulin was used as the loading control. (**H**,**I**) Fat body-specific *Hsf* knockdown reduced H3K4me3 enrichment within promoters of lipogenic genes and *Lpp* (**H**) as well as decreased the transcriptions of these lipid homeostasis-related genes (**I**) in the fat body. Data are presented as the mean ± SE (error bars). For the significance: * *p* < 0.05, ** *p* < 0.01, and *** *p* < 0.001 versus the control.

Given that transcription factors are generally required for mediating the binding of the COMPASS with histone methyltransferase activity to the promoter of target genes to catalyze H3K4me3 modification [34,35,36,37], we next sought to identify potential transcription factors that may mediate H3K4me3 modification at the lipogenic genes. First, based on in silico analysis of proteins that may interact with *Drosophila* Wds or its human homolog WDR5 and transcription factors that have potential binding sites within the promoters of lipogenic genes, 122 transcription factors were predicted to possibly both interact with Wds and bind to the promoters of lipogenic genes (Figure 4A and Table S6) according to gene expression data from the FlyBase database, 18 of which were expressed in the fat body (Figure 4A and Table S7). Further RNAi screen in the *Drosophila* fat body identified that only *Hsf* knockdown in the fat body could decrease LD size in the fat body and TG levels of *Drosophila* larvae (Figure 4B,C, Figure S5A,B and Figure S6A–D), but the knockdown of other transcription factors did not decrease LD size in the fat body (Figure S7). Moreover, fat body-specific *Hsf* knockdown also caused elevated TG levels and lipid accumulation in the intestine but decreased TG levels in the hemolymph (Figure 4D–F). These results phenocopied the effects of fat body-specific *Wds* knockdown, indicating that Hsf is involved in the regulation of lipid homeostasis in *Drosophila*. 

Next, we performed a Co-IP experiment using total proteins from *Drosophila* S2 cells co-overexpressing both Flag-tagged Hsf and 3xHA-tagged Wds. The result demonstrated that Hsf could be co-immunoprecipitated with Wds (Figure 4G). In addition, a ChIP-qPCR assay confirmed that the H3K4me3 enrichment within the promoters of lipogenic genes and *Lpp* could be significantly diminished by fat body-specific *Hsf* knockdown (Figure 4H). RT-qPCR examination showed that *Hsf* knockdown in the fat body reduced the expression of lipogenic genes and *Lpp* in the fat body (Figure 4I). Taken together, these data suggest that Hsf interacts with Wds to modulate H3K4me3 modification within the promoters of target genes to regulate lipid homeostasis in *Drosophila*.

### 2.5. HFD Condition Promotes Wds-Mediated H3K4me3 in the Drosophila Fat Body 

**Figure 5:**
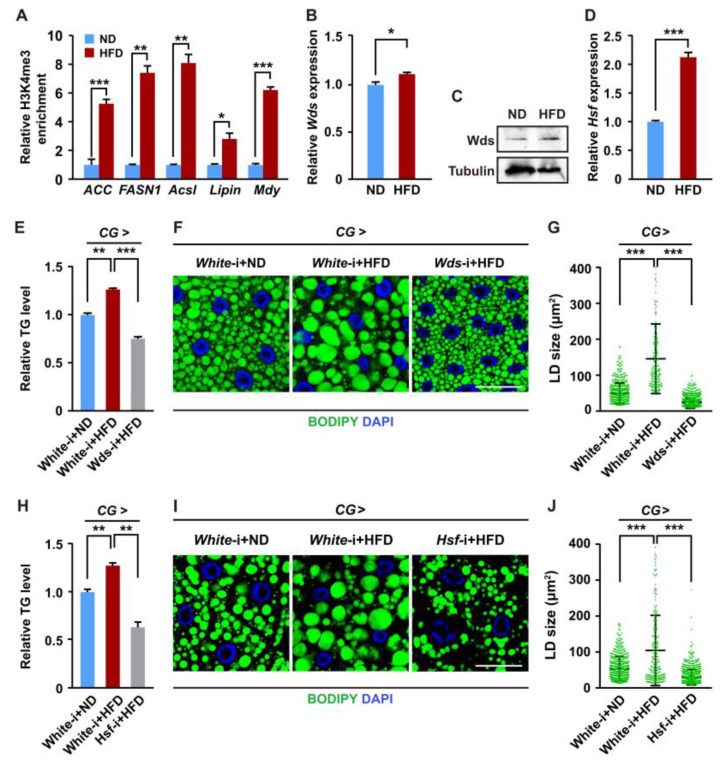
HFD increased the H3K4me3 levels to regulate lipid synthesis. (**A**) HFD feeding increased H3K4me3 levels within the promoters of lipogenic genes in the *Drosophila* fat body. (**B**) RT-qPCR confirmation of HFD-mediated upregulation in *Wds* mRNA expression in the fat body (n = 3, 10 larvae per group). (**C**) Western blotting confirmed the HFD-mediated increase in protein expression of Wds in the fat body. (**D**) RT-qPCR confirmation of HFD-mediated upregulation of the *Hsf* expression in the fat body (n = 3, 10 larvae per group). (**E**–**G**) Fat body-specific *Wds* knockdown could rescue HFD-induced defects in TG levels (n = 3, 10 larvae per group) of *Drosophila* larvae and LD size (**F**,**G**) in the fat body. Quantification of LD size in (**G**). Each point is a single LD. BODIPY, green; DAPI, blue. Scale bar, 50 μm. (**H**–**J**) Fat body-specific *Hsf* knockdown could rescue HFD-induced defects in TG levels (n = 3, 10 larvae per group) (**H**) of *Drosophila* larvae and LD size in the fat body. Each point is a single LD. BODIPY, green; DAPI, blue. Scale bar, 50 μm. ND, normal diet; HFD, high-fat diet. Data are presented as the mean ± SE (error bars). For the significance: * *p* < 0.05, ** *p* < 0.01, and *** *p* < 0.001 versus the control.

Increasing evidence has demonstrated that dietary HFD condition can increase lipid accumulation and TG levels [24,38,39,40]. Given that Wds-mediated H3K4me3 modification promotes the transcriptions of lipogenic genes to affect lipid accumulation in the *Drosophila* fat body, we therefore investigated whether HFD could regulate Wds-mediated H3K4me3 modification. We used a normal diet (ND) or HFD to feed *Drosophila* larvae at the third larval instar. First, immunostaining and western blotting assays found that HFD feeding elevated global H3K4me3 levels in the fat body compared with ND feeding as a control (Figure S8A,B). ChIP-qPCR analysis showed that H3K4me3 enrichment within the promoters of lipogenic genes was upregulated following HFD feeding (Figure 5A). Second, HFD feeding upregulated the expressions of the *Wds* gene in the fat body at mRNA and protein levels (Figure 5B,C and Figure S8C). *Hsf* expression in the fat body of *Drosophila* larvae was also upregulated by HFD feeding (Figure 5D). Importantly, the epistatic analysis revealed that HFD feeding-induced elevation of TG levels and LD size in the fat body were diminished by fat body-specific knockdown of *Wds* or *Hsf* (Figure 5E–J). Altogether, these data indicate that HFD promotes Wds-mediated H3K4me3 modification within promoters of lipogenic genes by upregulating the transcription of *Wds* and *Hsf*, thus contributing to the increase in lipid synthesis in the *Drosophila* fat body.

## 3. Discussion

Lipid homeostasis is determined by synthesis, transport, and mobilization and is crucial for insect growth and development [8,13,41,42,43]. Increasing evidence has revealed that numerous factors, including endocrine hormones, transcription factors, and microRNA, are involved in the regulation of lipid homeostasis [44,45]. H3K4me3 modification is epigenetically involved in transcriptional activation by relaxing the chromatin status at target loci and has been shown to be associated with various biological processes, such as stem cell proliferation and differentiation, development, memory formation, cancer, and autoimmune diseases [26,27,46,47]. A previous report on *Caenorhabditis elegans* has shown that H3K4me3 modification contributes to an increase in transgenerational lipid accumulation [33]. In the present study, by combing ChIP-seq and RNA-seq in *Drosophila* fat body, we demonstrate that Wds interacts with transcriptional factor Hsf to mediate H3K4me3 modification, which in turn directly targets lipogenic genes and *Lpp* to promote lipid synthesis and transport. Our findings provided novel insights into epigenetic regulation of lipid homeostasis during insect development and improved lipid metabolism network in insects.

An important finding of the present study is that Wds-mediated H3K4me3 modification epigenetically regulates lipid homeostasis in *Drosophila* by directly targeting most lipogenic genes associated with de novo lipid synthesis and the *Lpp* gene associated with lipid transport to promote their transcription. Notably, although previous studies have reported that lipoprotein Lpp mediates lipid transport from the intestine to hemolymph and then coordinates lipid distribution in the whole body to affect insect growth and development [18,48,49], the regulatory mechanism underlying *Lpp* transcription remains unclear. We demonstrated in *Drosophila* that fat body-specific *Wds* knockdown not only downregulated the *Lpp* expression but also disrupted lipid transport from the intestine, phenocopying the effects of fat body-specific *Lpp* knockdown on *Drosophila* lipid homeostasis [18]. Thus, our findings provide novel insight into the transcriptional regulation of genes involved in the maintenance of lipid homeostasis. 

Intriguingly, we also found in *Drosophila* that transcription factor Hsf can interact with Wds to mediate the H3K4me3 modification to regulate lipid homeostasis. Hsf has been reported to transcriptionally activate target genes from yeasts to humans [50,51]. There are two main *Hsf* genes in human, namely *Hsf1* and *Hsf2*. Only Hsf1 is characterized as a stress sensor. Hsf1 controls lipid metabolism in mouse adipose tissues by activating PGC1α expression and increasing mitochondrial function in muscle [52,53]. Under HFD dietary conditions, Hsf1 also enhances lipid expenditure by increasing the browning of white adipose tissue [54]. In contrast to this finding, *Hsf* has a single copy in *Drosophila,* and our study demonstrated that the knockdown of *Drosophila Hsf* in the fat body led to an obvious decrease in TG levels and LD size, indicating a key role of Hsf in lipid synthesis. In addition, human Hsf2 protein has been found to interact with the Set1/MLL complex to promote H3K4me3 modification, while Hsf1 protein has no effect on H3K4me3 modification [55]. Our results showed that Hsf interacted with Wds to elevate H3K4me3 levels to regulate lipid synthesis in *Drosophila*, indicating evolutionary conservation and diversity of Hsf action after the radiation of mammals and insects. The molecular mechanism underlying Hsf regulating the transcription of genes involved in lipid homeostasis remains to be determined. Furthermore, we predicted 122 transcription factors that potentially interact with Wds and bind to the promoters of lipogenic genes, but the functions of only 18 transcription factors in lipid synthesis have previously been explored. Thus, it will be of interest to investigate the rest of these transcription factors in the future.

## 4. Materials and Methods

### 4.1. Drosophila Cultivation and Stocks

*Drosophila* melanogaster lines were reared at 25 °C under a 12-h: 12-h light: dark cycle in a vail on standard food in a relative humidity of 70%. To enhance the RNA interference efficiency, *Drosophila* were reared at 29 °C for fat body-specific RNAi-mediated knockdown of transcription factors in this study. The normal diet (ND) food contained the following contents in one liter of H2O: 40 g sucrose, 42.4 g maltose, 5.5 g agar, 66.825 g yellow cornmeal, 9.2 g soy flour, 25 g dry yeast, 0.9 g p-hydroxybenzoic acid methyl ester (dissolved in 9 mL methanol), 1 g sodium benzoate, and 7 mL propionic acid. For the high-fat diet, the standard food was supplemented with 20% coconut oil (Sigma, St. Louis, MO, USA, #C1758) [24,56]. Approximately five fertilized female *Drosophila* lay eggs at 25 °C for 6 h. After hatching, the larvae firstly were reared with ND and transported into the HFD at the mid-third instar stage.

The CG-Gal4 and R4-Gal4 lines were used to specifically drive gene expression in the fat body [57]. The following RNAi stocks were obtained from the Vienna *Drosophila* Resource Center (VDRC): *UAS-Wds* RNAi (V105371) [58], *UAS-Hsf* RNAi (V37699) [59]. V60100 and V60000 lines were used as controls. *Drosophila* stocks from Bloomington *Drosophila* Stock Center (BDSC) include CG-Gal4 (BS7011), R4-Gal4 (BS33832), and *UAS-Wds* RNAi (BS32952). *Drosophila* stocks from TsingHua Fly Center include *UAS-Hsf* RNAi (THU2458), *UAS-Dp* RNAi(THU4866), *UAS-HLHmβ* RNAi (THU2201), *UAS-Luna* RNAi (THU2473), *UAS-Cyc* RNAi (THU5935), *UAS-Ci* RNAi (TH01945.N), *UAS-Arm* RNAi (THU1631), *UAS-Slob* RNAi (TH02008.N), *UAS-P53* RNAi (THU2533), *UAS-Vvl* RNAi (THU2227), *UAS-Trl* RNAi (THU4158), *UAS-Tgo* RNAi (THU2366), *UAS-D* RNAi (THU2216), *UAS-Sox14* RNAi (THU0640), *UAS-Clamp* RNAi (TH03528.N), *UAS-Cdc5* RNAi (TH02910.N), *UAS-CrebB* RNAi (THU2514), and *UAS-Sima* RNAi (TH201501074.S). *UAS-White* RNAi (THU0558) line was used as a control.

### 4.2. Immunostaining

The procedure for immunostaining has been described in our previous report [60]. In brief, the fat body tissues of *Drosophila* larvae at the third instar stage were dissected in PBS and fixed with 4% paraformaldehyde for 30 min at room temperature. After being washed with 0.3% PBST (PBS with 0.3% Triton-X 100) three times, the tissues were incubated at 4 °C overnight with anti-H3K4me3 antibodies (1:500, Abcam, Cambridge, UK, #ab8580) [61]. Then the tissues were washed with 0.3% PBST buffer for three times and incubated with goat anti-rabbit Alexa Fluor 594 (1:1000, Life Technologies, Santa Clara, CA, USA, #R37117) for 1 h at room temperature. The cell membrane was stained with Alexa Fluor 488-phalloidin (1:500, Invitrogen, Carlsbad, CA, USA, #A12379) for 1 h at room temperature. After being washed with 0.3% PBST buffer, the cell nuclei of samples were stained with DAPI (1:1000, Thermo Fisher Scientific, Carlsbad, CA, USA, #D1306) for 30 min at room temperature. Finally, the fat body tissues were mounted in Vectashield mounting buffer after being washed three times with PBS buffer. For LD staining, the fat bodies were stained with BODIPY™ 493/503 (1 mg/mL, Invitrogen, Carlsbad, CA, USA, #D3922) [21] and DAPI for 30 min at room temperature. The fluorescence signals were captured by a confocal microscope (Zeiss, Oberkochen, Germany, LSM 880). The size of LDs was quantified by Fiji software. The LDs were outlined using the Analyze Particles option in the Fiji software, and the LD area was further measured according to the scale bar.

### 4.3. RNA-seq

Fat bodies of 20 *Drosophila* larvae with *White* RNAi or *Wds* RNAi were dissected and collected in quadruplicate, respectively. The total RNA was extracted using Trizol reagent (Invitrogen, Carlsbad, CA, USA) and purified on RNAeasy columns (QIAGEN, Dusseldorf, Germany) to facilitate subsequent RNA-seq. The *Drosophila* reference genome was downloaded from FlyBase (http://ftp.flybase.net/genomes/, 25 October 2021). Clean reads were mapped to the *Drosophila* reference genome quickly and accurately using HISAT2(v2.0.5) software [62]. The featureCounts v1.5.0-p3 was used to count the number of reads mapped to each gene. Subsequent analyses for RNA-seq were based on uniquely mapping data. The FPKM (fragments per kilo base of transcript per million mapped fragments) method was used to normalize gene expression. DEG (fold change >= 1.5 and padj < 0.05) was performed using the DESeq2 R package (1.20.0). 

### 4.4. Cell Culture and Transfection 

*Drosophila* embryonic Schneider 2 (S2) cells were cultured in Schneider *Drosophila* medium (Gibco, Carlsbad, CA, USA, 21720024) supplemented with 10% fetal bovine serum (FBS) and 1% antibiotics of penicillin and streptomycin at 27 °C in 5% CO2. The open reading frame (ORF) sequence of the *Drosophila Wds* and *Hsf* genes were respectively subcloned into pMT-V5-HisA vector for gene overexpression in *Drosophila* S2 cells. A pair of primers against *Wds* is as follows: 5′-ATGGTGCGCTCCTCCAAGAAC, 5′-CTACAGGAACAGGTGGTGGCG. The primer pair for *Hsf* is as follows: 5′-TCCAGGTCGCGTTCA, 5′-CAACTCGTGACGTGGCGT. Then, these two plasmids were co-transfected into S2 cells, and 500 μM CuSO4 was added after 6 h to induce the S2 cells to stably express the HA-tagged Wds and Flag-tagged Hsf proteins. After transfection for 2.5 days, the S2 cells were collected for subsequent co-immunoprecipitation.

### 4.5. Co-Immunoprecipitation (Co-IP)

For Co-IP analysis, the co-transfected S2 cells were lysed in NP-40 lysis Buffer (Beyotime, Shanghai, China, #P0013F) containing 1mM phenylmethanesulfonyl fluoride (PMSF) (Beyotime, Shanghai, China, #ST506) for 10 min on ice. Next, the cells were centrifuged at 13,000 rpm for 10 min at 4 °C and then transferred supernatant to a new microfuge tube. Dynabeads protein A for immunoprecipitation (DPA beads) (Invitrogen, Carlsbad, CA, USA, #10001D) were firstly washed with NP-40 lysis buffer three times. HA-tag rabbit antibodies (1:50, Cell Signaling Technology, Danvers, MA, USA, #3724) were incubated with DPA beads containing 5 mM of BS3 [bis (sulfosuccinimidyl) suberate] (Thermo Fisher Scientific, Carlsbad, CA, USA, #21580) under gentle rotation for 6 h at 4 °C. Rabbit IgG (Cell Signaling Technology, Danvers, MA, USA, #2729) was used as a negative control. Then, the beads were washed with NP-40 lysis buffer three times. The target proteins subsequently were eluted with SDT buffer (100 mM Tris/HCl pH 7.4, 4% SDS). Equal amounts of proteins from immunoprecipitated products with a 10 min boil were further subjected to western blotting. 

### 4.6. Western Blotting 

Total proteins from S2 cells or fifteen *Drosophila* larval fat body tissues that were dissected at late third-instar larval stage lysed by NP-40 lysis buffer (Beyotime, #P0013F) containing 1 mM PMSF (Beyotime, Shanghai, China, #ST506). After being extracted, the supernatant was collected, and quantification of the protein concentration was performed using an enhanced BCA protein assay kit (Beyotime, Shanghai, China, #P0009). Equal proteins were loaded on 15% SDS-PAGE gels and then transferred the target protein to PVDF membranes (BioRad, Hercules, CA, USA, #1620219). The membranes were blocked with QuickBlock™ blocking buffer (Beyotime, Shanghai, China, #P0231) at room temperature for 15 min. The following primary antibodies were used to incubate membranes at 4 °C overnight: anti-H3K4me3 (1:1000, Abcam, Cambridge, UK, #ab8580), anti-H3 (1:1000, Cell Signaling Technology, Danvers, MA, USA, #9715s) or anti-Wds (1:1000, Abcam, Cambridge, UK, #ab178410). The second set of antibodies, HRP-conjugated goat anti-rabbits (1:10,000, Beyotime, Shanghai, China, #A0208), was used to incubate membranes for 1 h at room temperature. The corresponding protein bands were detected using Pierce™ ECL Western blotting substrate kit (Thermo Fisher Scientific, Carlsbad, CA, USA, #2209). 

### 4.7. Chromatin Immunoprecipitation Sequencing (ChIP-seq)

Fat bodies of about 600 third-instar larvae were dissected in PBS and collected to cross-link proteins to DNA with 37% paraformaldehyde (Sigma, St. Louis, MO, USA, #252549) for 20 min at room temperature. Next, a SimpleChIP^®^ Plus enzymatic chromatin IP kit was used (Magnetic Beads) (Cell Signaling Technology, Danvers, MA, USA, #9005s) to carry out the ChIP experiment with antibodies for H3K4me3 (Abcam, Cambridge, UK, #ab8580) according to the manufacturer’s instructions. Briefly, after stopping cross-linking with glycine, a Dounce homogenizer was used to disaggregate tissues into a single-cell suspension. Then, the suspension was collected, cells were collected immediately for nuclei preparation and chromatin digestion. Afterward, DNA was digested to lengths of approximately 150–900 bp and sonicated with up to 500 µL of lysate to break nuclear membranes with 5 min pulse s on wet ice until lysis of the nuclei was complete. The lysates were centrifugated at 10,000 rpm for 10 min at 4 °C, and the supernatant was collected for the chromatin immunoprecipitation preparation. 2 µg of H3K4me3 antibodies were added into IP sample and incubated overnight at 4 °C under slow rotation. An equal amount of rabbit IgG (Cell Signaling Technology, Danvers, MA, USA, #2729) was used as the negative control, and 150 µL of the chromatin preparation was removed as the input and stored at −80 °C until further use. 30 µL of Protein G magnetic beads were added into each IP sample, and the mixtures were incubated for 2 h at 4 °C with slow rotation. After the elution of chromatin from the antibody/protein G magnetic beads, the DNA fragments were purified using Dr. Gen TLE™ Precipitation Carrier (Takara, Kyoto, Japan, #9094). 

For ChIP-sequencing, DNA fragments were purified from each eluted sample and sequenced via Illumina HiSeqTM by the Novagene (Beijing, China). The quality of the raw reads was assessed using FastQC (v0.11.5) and reads mapped onto the *Drosophila* reference genome using a BWA (Burrows Wheeler Aligner). Using MACS2 (v2.1.0), the aligned reads were then subjected to peak calling with a q-value <= 0.05. ChIPseeker was used to retrieve the nearest genes around the peak. IGV software was used to visualize the ChIP-seq data and distinguish the sites of histone methylation. Compared to the input sample, the reads with a two-fold enrichment were considered as difference peaks. GO enrichment and KEGG pathway enrichment assays were implemented by the GOseq R package and KOBAS software, respectively. The H3K4me3 occupancy within promoters for indicated lipogenic genes and *Lpp* gene was confirmed by chromatin immunoprecipitation following basic PCR or RT-qPCR (ChIP-PCR and ChIP-qPCR). The fold enrichment values of ChIP-qPCR experiment were normalized to the input. All specific primers used for ChIP-PCR and ChIP-qPCR are listed in Table S8. 

### 4.8. RNA Extraction and Real-Time Quantitative PCR (RT-qPCR)

Trizol reagent (Invitrogen) was used to extract larval fat body total RNA. cDNAs were synthesized using 1 µg total RNA according to the protocol of EasyScript one-step gDNA removal and cDNA synthesis super mix kits (TransGen, Bejing, China). RT-qPCR was performed with a NovoStart ^®^ SYBR qPCR super mix plus (Novoprotein, Suzhou, China) by using a 7500 fast real-time PCR System (Applied Biosystems, Carlsbad, CA, USA). The ribosomal protein 49 gene (*RP49*) was used as the internal control gene. All primers used for RT-qPCR are listed in Table S8. All experiments were independently performed with three biological replicates, and the relative mRNA expression levels were calculated using the 2^−ΔΔCT^ method.

### 4.9. TG Measurement

TG measurement was performed as previously described [63]. Ten larvae at the third instar stage were washed with PBS, collected in the 1.5 mL microfuge tube, and put into the liquid nitrogen for quick freezing. Then, the *Drosophila* were homogenized in the 350 µL PBS with 0.5% Triton-X 100, supplemented with 1 mM PMSF (Beyotime, Shanghai, China, #ST506). For measurement of hemolymph TG, hemolymph from 60 3rd instar larvae was diluted in 100 µL PBS per simple. Larvae homogenate or hemolymph was then heated at 70 °C for 5 min to deactivate endogenous enzymes and centrifuged at 13,000 rpm for 15 min. The supernatant was used to measure TG using serum TG determination kits (Sigma, St. Louis, MO, USA, #TR0100). The protein amounts were measured using an enhanced BCA protein assay kit (Beyotime, Shanghai, China, #P0009). TG levels were normalized to the protein amounts. All experiments were independently performed with three biological replicates. 

### 4.10. Databases

The transcription factors binding at the promoters of the lipogenic gene were predicted by AnimalTFDB3.0 (http://bioinfo.life.hust.edu.cn/AnimalTFDB, 28 February 2022). Wds/WDR5 interacting proteins were predicted using bioGRID (https://thebiogrid.org/, 28 February 2022). 

### 4.11. Statistical Analysis

We used a Student’s t-test for comparisons between the two groups. For comparisons of LD size, we used a one-way ANOVA with a post hoc Dunnett test. Data are presented as the mean ± SE (error bars). For the significance test: * *p* < 0.05, ** *p* < 0.01, and *** *p* < 0.001 versus the control.

## 5. Conclusions

Wds-mediated H3K4me3 modification in the *Drosophila* fat body directly targets lipogenic genes and *Lpp* to promote lipid synthesis and transport through the transcriptional factor Hsf. How Hsf participates in regulating the transcription of genes involved in lipid homeostasis remains to be determined.

## Data Availability

All the raw RNA and ChIP sequence data have been deposited in the National Center for Biotechnology Information (NCBI) Short Read Archive (http://www.ncbi.nlm.nih.gov/ sra/, 26 October 2022 and 8 November 2022) under the Sequence Read Archive (SRA) accession number (PRJNA894775 and PRJNA909973).

## Supplementary Materials

The following supporting information can be downloaded at: https://www.mdpi.com/article/10.3390/ijms24076125/s1.
