# Supplementary material for: Wds-Mediated H3K4me3 Modification Regulates Lipid Synthesis and Transport in Drosophila
Source: Int J Mol Sci. 2023 Mar 24;24(7):6125. doi: 10.3390/ijms24076125 (PMC10093852; doi:10.3390/ijms24076125)
Supplement: Supplementary file 1 [file ijms-24-06125-s001.zip › ijms-2251742-supplementary/Supporting Information/ijms-2251742-Supplementary figures.pdf]

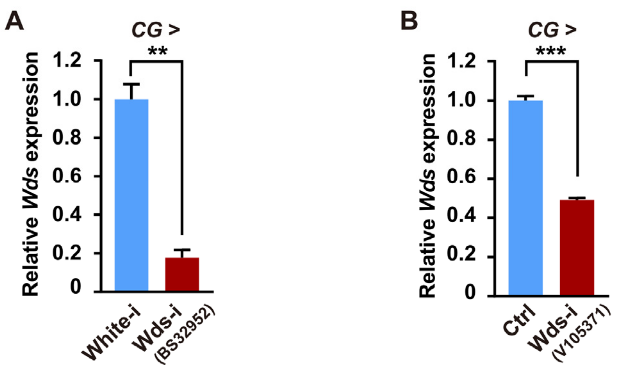

Supplementary Figure 1. Fat body-specific *Wds* knockdown decreased *Wds* expression in the fat body. (A) Fat body-specific *Wds* knockdown using TRiP line (BS32952) decreased the *Wds* expression in the fat body (n = 3, 10 larvae per group). This genetic manipulation for *Wds* was used here after unless otherwise indicated. **(B)** Fat body-specific *Wds* knockdown using VDRC line (V105371) decreased the *Wds* expression in the fat body (n = 3, 10 larvae per group). Data are presented as the mean ± SE (error bars). For the significance: ** $p < 0.01$, and *** $p < 0.001$ versus the control.

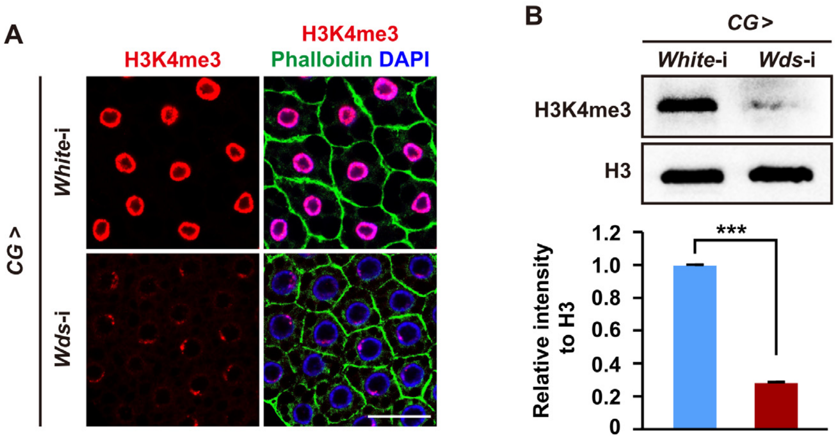

Supplementary Figure 2. Fat body-specific *Wds* knockdown decreased H3K4me3 levels. (A-B) TRiP RNAi line (BS32952)-mediated *Wds* knockdown decreased global H3K4me3 levels in the fat body, which was shown by immunostaining (A) and western blotting (B). H3K4me3, red; phalloidin, green; DAPI, blue. Scale bar, 50 μm. Western blot data of the H3K4me3 levels was quantified (B). Data are presented as the mean ± SE (error bars). For the significance: *** $p < 0.001$ versus the control.

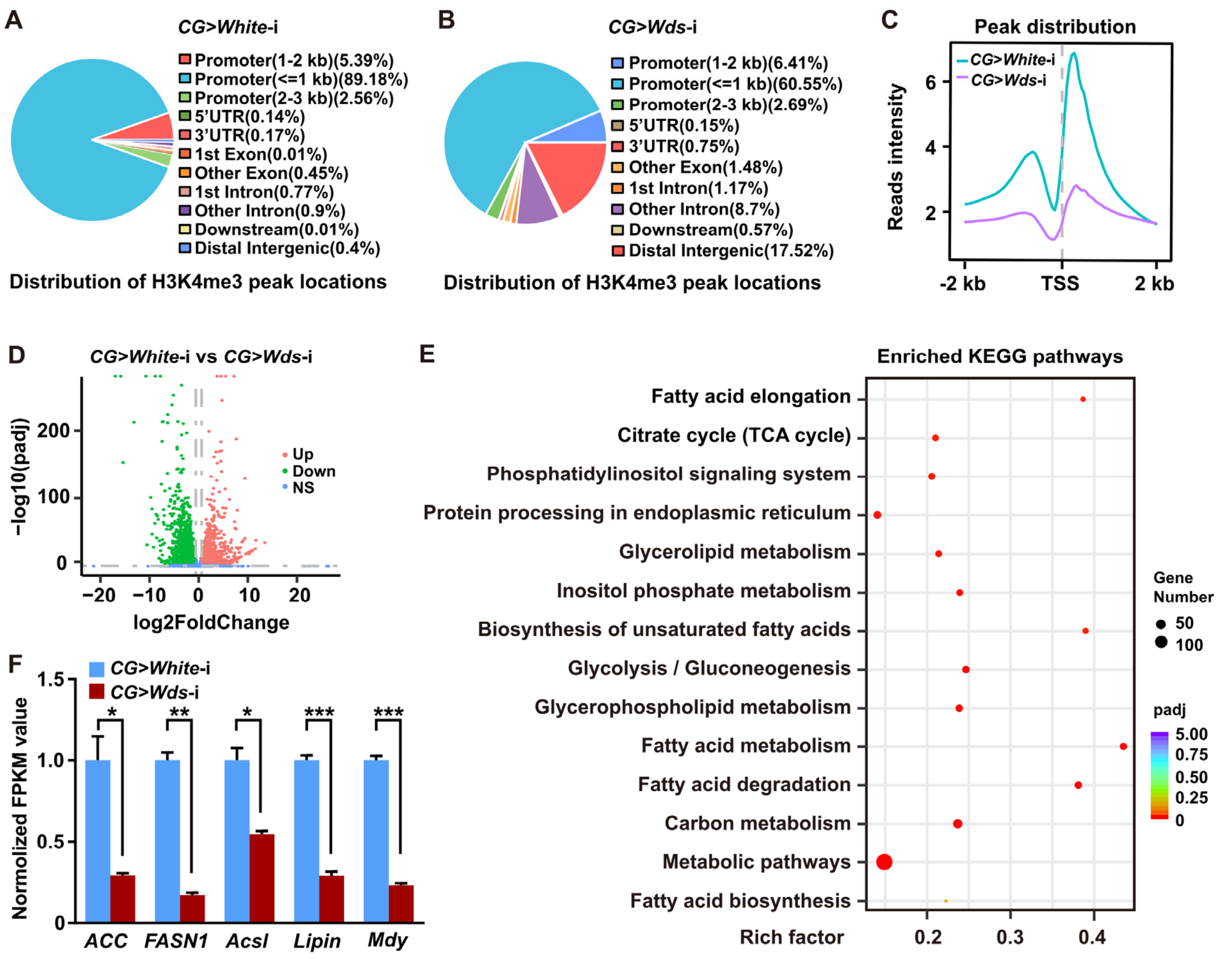

Supplementary Figure 3. ChIP-seq and RNA-seq-based identification of potential targets for Wds-mediated H3K4me3 modification in the *Drosophila* fat body. (A-B) Fat body-specific *Wds* knockdown-caused changes in genomic location of H3K4me3 ChIP peaks in the fat body. **(C)** ChIP-seq identified the change of H3K4me3 enrichment around genome-wide transcription start sites (TSSs) in the fat body of *Drosophila* larvae at 120 h AEL following fat body-specific *Wds* knockdown. The density plot of H3K4me3 ChIP peaks around the TSSs were constructed. **(D)** Volcano plot for fat body-specific *Wds* knockdown-caused change in gene expression in fat body. **(E)** Scatter plot of the enriched metabolic processes-related KEGG pathways for all genes with downregulated H3K4me3 ChIP peaks (depDEGs). The size and color denote gene number in each KEGG pathway and significant enrichment; rich factor is calculated by using the depDEG number to divide the number of

all genes in same pathway. **(F)** RNA-seq data of *Wds* knockdown-induced decrease in mRNA expression of lipogenic genes in *Drosophila* larval fat body. FPKM, fragments per kilo base of transcript per million mapped fragments. Data are presented as the mean ± SE (error bars). For the significance: * $p < 0.05$, ** $p < 0.01$, and *** $p < 0.001$ versus the control.

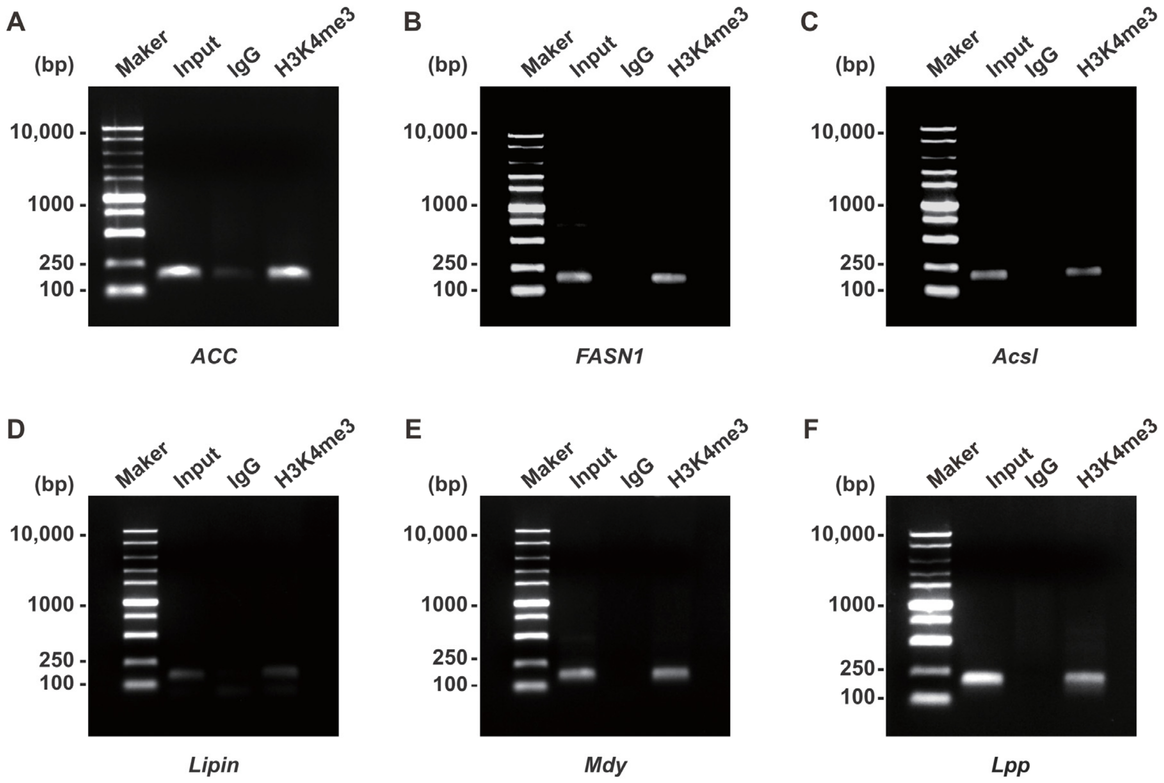

Supplementary Figure 4. ChIP-PCR assay of H3K4me3 deposition in the DNA regions covering the H3K4me3 ChIP peaks within the promoters of several lipogenic genes and the *Lpp* gene. (A) H3K4me3 deposition in the DNA region covering the H3K4me3 ChIP peak within *ACC* promoter. **(B)** H3K4me3 deposition in the DNA region covering the H3K4me3 ChIP peak within *FASN1* promoter. **(C)** H3K4me3 deposition in the DNA region covering the H3K4me3 ChIP peak within *Acsl* promoter. **(D)** H3K4me3 deposition in the DNA region covering the H3K4me3 ChIP peak within *Lipin* promoter. **(E)** H3K4me3 deposition in the DNA region covering the H3K4me3 ChIP peak within *Mdy* promoter. **(F)** H3K4me3 deposition in the DNA region covering the H3K4me3 ChIP peak within *Lpp* promoter.

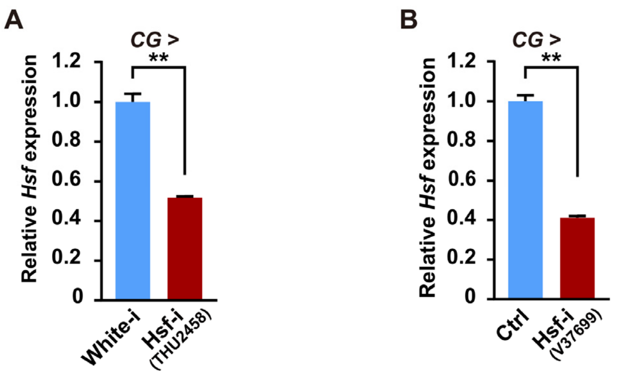

Supplementary Figure 5. Fat body-specific *Hsf* knockdown decreased *Hsf* expression in the fat body. (A) Fat body-specific *Hsf* knockdown using TRiP line (THU2458) decreased the *Hsf* expression in the fat body (n = 3, 10 larvae per group). **(B)** Fat body-specific *Hsf* knockdown using VDRC line (V37699) decreased the *Hsf* expression in the fat body (n = 3, 10 larvae per group). Data are presented as the mean ± SE (error bars). For the significance: ** $p < 0.01$ versus the control.

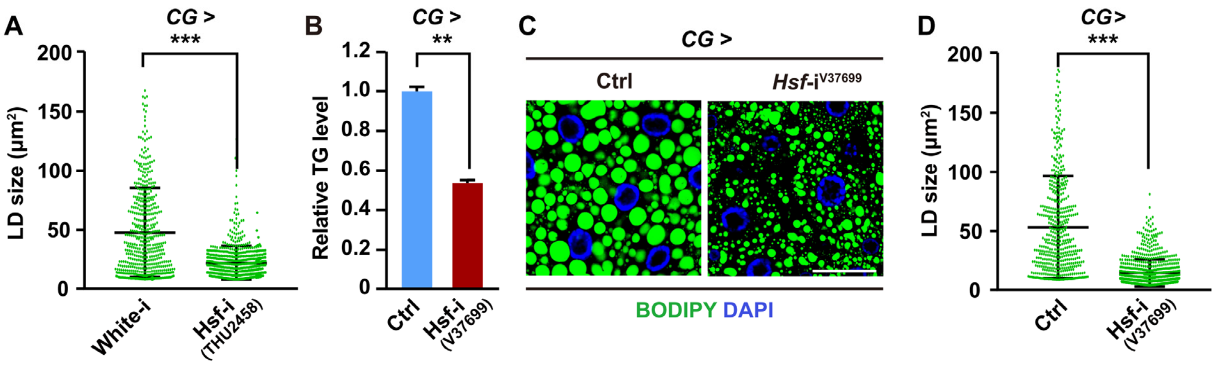

Supplementary Figure 6. Fat body-specific *Hsf* knockdown decreased lipid content. (A) Quantification of TRiP RNAi line (THU2458)-mediated *Hsf* knockdown-caused decrease in LD size in the fat body of *Drosophila* third instar larvae, related to Figure 4B. Each point represents a single LD. **(B–D)** VDRC RNAi line (V37699)-mediated *Hsf* knockdown decreased TG levels (n = 3, 10 larvae per group) (B) of *Drosophila* third instar larvae and LD size (C–D) in the fat body. V60000 line was used as control. Each point represents a single LD. BODIPY, green; DAPI, blue. Scale bar, 50 μm. Data are presented as the mean ± SE (error bars). For the significance: ** $p < 0.01$, and *** $p < 0.001$ versus the control.

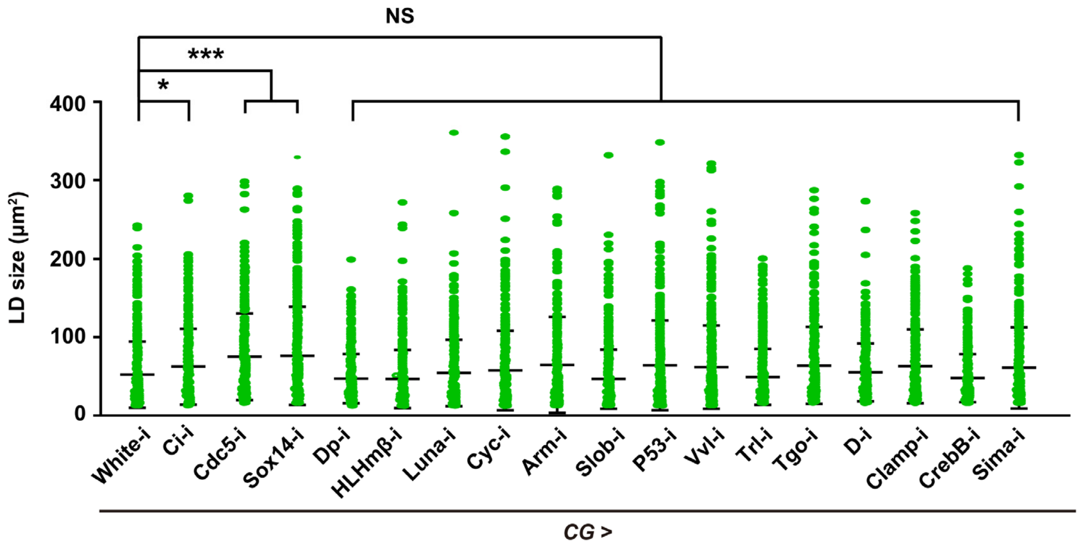

Supplementary Figure 7. The effect of fat body-specific knockdown of selected transcription factor genes on LD size. Fat body-specific knockdown of selected transcription factor genes, including *Ci*, *Cdc5*, *Sox14*, *Dp*, *HLHmβ*, *Luna*, *Cyc*, *Arm*, *Slob*, *P53*, *Vvl*, *Trl*, *Tgo*, *D*, *Clamp*, *CrebB*, or *Sima*, did not decrease LD size in the fat body of *Drosophila* third instar larvae. Each point represents a single LD. BODIPY, green; DAPI, blue. Scale bar, 50 μm. Data are presented as the mean ± SE (error bars). For the significance: * $p < 0.05$, and *** $p < 0.001$ versus the control.

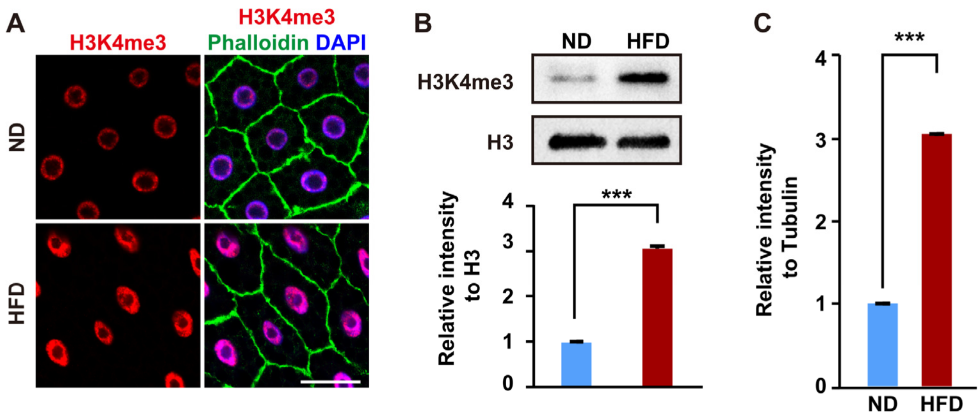

Supplementary Figure 8. HFD upregulated H3K4me3 levels and Wds protein levels. (A-B) HFD feeding increased H3K4me3 levels globally in the *Drosophila* fat body. ND, normal diet; H3K4me3, red; phalloidin, green; DAPI, blue. Scale bar, 50 μm. Western blot data of H3K4me3 levels was quantified (B). **(C)** Western blot data of Wds protein levels was quantified. Data are presented as the mean ± SE (error bars). For the significance: *** $p < 0.001$ versus the control.
